# Dietary Oleic Acid and SCD16 and ELOVL6 Estimated Activities Can Modify Erythrocyte Membrane n-3 and n-6 HUFA Partition: A Pilot Study

**DOI:** 10.3390/cimb47020081

**Published:** 2025-01-27

**Authors:** Paulo Bispo, Pedro O. Rodrigues, Narcisa M. Bandarra

**Affiliations:** 1 Department of Food Tecnhology, Biotecnology and Nutrition, Escola Superior Agrária, Instituto Politécnico de Santarém, 2001-904 Santarém, Portugal; 2 Department of Biochemistry and CEDOC, Faculty of Medical Sciences/NOVA Medical School, Universidade Nova de Lisboa (UNL), 1169-056 Lisboa, Portugal; prof.orlandorod@gmail.com; 3 IPMA, IP, Departamento do Mar e dos Recursos Marinhos, Divisão de Aquacultura, Valorização e Biosprospeção, 1495-165 Lisboa, Portugal; narcisa@ipma.pt; 4 CIIMAR, Centro Interdisciplinar de Investigação Marinha e Ambiental, Terminal de Cruzeiros de Leixões, 4450-208 Matosinhos, Portugal

**Keywords:** dietary oleic acid, n-6/n-3 HUFA, metabolism, cardiometabolic biomarkers, red blood cells

## Abstract

In this work, we studied the relationships between the most representative fatty acids (FAs) and their ratios in red blood cell (RBC) membranes and dietary fatty acids alongside several cardiometabolic risk factors. Twenty-six individuals were enrolled with a mean age of 50.4 ± 12.7 years (16 males and 10 females). By bivariate analysis, dietary oleic acid (OA) correlated negatively with C20:4n-6 (AA) (*p* = 0.031) in RBCs. With multivariate regression analysis, dietary OA (*p* < 0.001) is an independent predictor and negatively associated with AA levels in RBCs, while the elongation of very-long-chain fatty acids 6 (ELOVL6) and stearoyl-CoA desaturase 16 (SCD16) activities (*p* < 0.05) was positively associated with AA levels in RBCs. The multivariate regression models also showed that dietary OA was an independent predictor and positively associated with C22:5n-3 (DPA) in RBCs. Furthermore, BMI positively correlated with SCD16, and both SCD16 and SCD18 were positively associated with triacylglycerols levels. In addition, SCD16 positively and significantly correlated with LDL-c and the LDL-c/HDL-c ratio and negatively correlated with the ApoA1/ApoB ratio, and SCD16 and ELOVL6 were significantly associated with HDL molecular subfractions. Therefore, our data underline that OA, SCD16 and ELOVL6 can interfere with n-3 and n-6 partition in biomembranes such as RBCs, suggesting an important molecular (patho)physiological regulatory mechanism role in controlling bioactive molecules’ availability such as those involved in the immune-inflammatory response.

## 1. Introduction

The red blood cell (RBC) membrane’s fatty acid profiles have been receiving increasing attention as a biomarker not only of food intake but also as a hallmark of metabolic status and the prevention of cardiovascular disease (CVD).

In fact, dietary fatty acids reflect partially the fatty acid profile of biological systems, and numerous variables are involved in fatty acid bioavailability and absorption. Digestion, absorption, and metabolic pathways (e.g., desaturase or elongase activity), as well as the storage and exchange amongst compartments, greatly influence fatty acids’ composition in plasma and several blood and tissues cells [1]. Industrialized societies are characterized by an increase in energy intake and decrease in energy expenditure, by an increase in saturated fat and n-6 fatty acids, and by a decrease in n-3 fatty acid intake as well. Excessive amounts of dietary n-6 fatty acids (such as linoleic acid, LA) increase the enzymes’ metabolic cascade for the desaturation (delta-5 and delta-6 desaturases) and elongation. A very high n-6/n-3 ratio, as is found in today’s Western diets, has been shown to promote the pathogenesis of many diseases, including CVD, cancer, metabolic inflammation and autoimmune diseases, whereas increased levels of n-3 PUFAs (a lower n-6/n-3 ratio) exert suppressive beneficial effects [2,3]. A number of studies have emphasized the influence of FA composition in different pathophysiological settings, such as those involved in insulin resistance, eicosanoids production and signaling pathways. It has been demonstrated that changes in fatty acids profile were common features observed in the metabolic syndrome, obesity, diabetes and CVD [4,5,6,7]. Dietary strategies are accepted as a major factor in the prevention of those diseases. However, conflicting recommendations exist concerning the nature and amount of different dietary fats, fatty acids, and the ratios among them [8,9].

Fatty acids are highly regulated “in vivo”, and the cross-talk between the biochemical regulation of saturated fatty acids (SFAs) and monounsaturated fatty acids (MUFAs) might influence the bioavailability of polyunsaturated fatty acids (PUFAs), e.g., in lipid classes such as phospholipids (PLs), and vice a vice (vv). In this respect, several proteins, such as long-chain acyl-coenzyme A synthetases (e.g., ACSL4), fatty acid binding proteins (FABP) or lysophospholipid acyltransferases (e.g., LPCAT3); and transcription factors, like sterol regulatory element-binding protein (SREBP), carbohydrate-responsive element-binding protein (ChREBP) or peroxisome proliferator-activated receptors (PPARs), e.g., alpha (PPARα) have a pivotal role in the fatty acids profile of different phospholipid classes, by regulated, e.g., both the novo pathway (Kennedy pathway) and the remodeling pathway (Lands pathway). Lipogenic enzymes, such as fatty acid synthase (FAS), involved in the “de novo” lipogenesis (DNL), and stearoyl-CoA desaturase (SCD) are key enzymes in lipid and energy metabolism that greatly affect the SFA/MUFA composition of a membrane’s PL [10,11,12,13]. It is well known that desaturases (e.g., SCD1) and elongases (e.g., ELOVL6) are essential enzymes in regulating the degree of unsaturation and length of fatty acids and, inherently, influence cellular functions and signaling pathways as well as their metabolic fates (e.g., integration in biomembranes or for β-oxidation) [14,15,16,17]. In addition, several fatty acids can modulate the expression of genes associated with, e.g., SCD1, and in relation to the regulation of SCD1 via SREBP-1c the effect of C22:6 n-3 (DHA) appears to be superlative compared to other PUFA of n-3 and n-6 families. Despite the presences of several isoforms with tissue and substrate specificity, SCD1 is an isoform mainly found in liver and adipose tissue. Interestingly, SCD1 seems to act as a “*double-edge sword*” because it is involved in both obesity and hypertriacylgliceridemia (if up-regulated) or atherosclerosis and inflammation (if down-regulated) [11].

In the present pilot study, we evaluated the correlation between the fatty acid in red blood cell (RBC) membranes and the dietary fatty acids composition as well as some cardiometabolic biomarkers. To our knowledge, few research papers were conducted applying, simultaneously, dietary data, RBC fatty acids profile, and cardiometabolic biomarkers, like HDL molecular subfractions. Based on the obtained data, we tried to elucidate a few metabolic mechanisms involved in the effect of dietary oleic acid (OA) in the bioavailability of highly unsaturated fatty acids (HUFA) n-3 and n-6, which in turn can be related with consumer recommendations associated with the prevention of coronary heart disease (CHD) in the case of OA-derived MUFA rich diets.

## 2. Subjects and Methods

**Participants.** The sample population comprised twenty-six consecutive free-living individuals from both genders (16 male and 10 female) with age ≥ 18 years. The volunteers were recruited following contacts, which began in 2009, with the medical community living in public hospitals, and in particular with outpatient clinics, as well as a private clinic in the Greater Lisbon region, together with magazine advertisements and verbal contacts/invitations at the University Campus of UNL (Lisbon) and ESTM-IPL (Peniche). This work is part of the baseline data of a nutritional intervention study. The study began in April 2010 and ended in April 2012. Exclusion factors included pregnant or breastfeeding women and the presence of cancer and/or treatment for (in the last 6 months). In the present study, we excluded participants (*n* = 5) who took nutritional supplements. The subjects were assessed according to the same clinical, biochemical and anthropometric protocol, and data were collected. Informed consent was obtained from all participants. The experimental protocol was approved by the Ethical Committee of Western Lisbon Hospital Center (EC 196/2008, 6 May 2008), Portugal, in accordance with the Helsinki Declaration.

**Blood biochemical assays.** Venous blood was sampled after an overnight fast and the plasma analyzed for glucose, triacylglycerols (TAGs), total cholesterol (Total-chol), high-density lipoproteins cholesterol (HDL-c), ApoA1, ApoB and high sensitivity C-reactive orotein (hsCRP), which were obtained by enzymatic and turbidimetric techniques (Roche Diagnostics, Mannheim, Germany) using a Hitachi 911 (Hitachi, Tokyo, Japan). The ApoA1/ApoB ratio was calculated. HDL molecular subfractions were determined using a polyacrylamide gel electrophoresis system LIPOPRINT (Quantimetrix, Redondo Beach, CA, USA) according to the manufacturer’s instructions [18] in a subset of the population sample (*n* = 16). HDL subfractions (*n* = 10) were quantified and grouped into large HDL (L-HDL), intermediate HDL (I-HDL) and small HDL (S-HDL) and then expressed in percentage (%) and quantity (mg/dL).

**Dietary record.** Dietary intake was obtained using semi-quantitative food frequency questionnaire, validated for the Portuguese population, comprising 82 items, and reported to the previous year. Food consumption was converted into total energy intake and nutrients using the software Food Processor Plus (version 5.0, ESHA Research, Salem, OR, USA), which was adapted to traditional Portuguese food items as previously described [19,20]. Dietary fatty acids were evaluated as a percentage of total fat in diet.

**RBC membranes fatty acid profile.** Blood was obtained by venous puncture, and 5 mL of heparinized blood was left to rest for about 30 min; red blood cells were obtained by centrifuging for 10 min at 700× *g*, discarding the supernatant, washing the pellet twice with 0.9% NaCl, and centrifuging again for 5 min at 700× *g*. The obtained pellet (0.5 mL) was freeze dried and stored at –80 °C for further analysis. Phospholipids’ membrane fatty acid methyl esters (FAMEs) were obtained through the addition of 1 mL of anhydrous methanol, 0.5 mL of sodium methoxide (1 M in methanol), swirling for 5 min, and 1 h reaction in the dark, following the conditions referred by [21]. The layer separation was improved through 10 min in an ultrasonic bath and 5 min centrifuging at 1500× *g*. The n-hexane layer was collected and the aqueous phase re-extracted with 2.5 mL of n-hexane and centrifuging again. FAME were concentrated to a final volume of 25 μL in n-heptane and 2 μL of sample was injected on a capillary DB-Wax capillary column (30 m, 0.25 mm internal diameter and 0.25 μm film thickness, Agilent J&W Scientific, Santa Clara, CA, USA) in a Varian CP-3800 gas chromatograph equipped with a flame ionization detector (Varian, Palo Alto, CA, USA). The injector and detector temperature were set at 250 °C. Adequate separation was obtained over a 40-min period of time: 5 min at 180 °C, a ramp temperature increase of 4 °C/min until 220 °C, and 25 min at 220 °C. FAME identification was obtained through comparison with high purity standards from Sigma (Sigma, St. Lois, MO, USA). The relative percentage amount of fatty acids in RBC membranes was determined, and the C16:1/C16:0, C18:1/C18:0 and C18:0/C16:0 ratios were calculated. The first two ratios give product-to-precursor measures of the stearoyl-CoA-desaturase (SCD or D9D), SCD16 and SCD18, respectively. The C18:0/C16:0 ratio evaluated the elongase ELOVL6 activity.

**Statistical analysis.** Descriptive, Spearman’s correlation coefficient and partial correlations adjusted for age, Body Mass Index (BMI) and total energy intake were determined (SPSS Inc., Chicago, DE, USA). Kurtosis, Shapiro–Wilk and/or Kolmogorov–Smirnov tests for normality were performed, and skewed distribution variables were log-transformed. Multivariate regression analysis (stepwise method) was conducted with dietary palmitic acid (PA), stearic acid (SA), oleic acid (OA), linoleic acid (LA), arachidonic acid (AA), eicosapentaenoic acid (EPA), docosahexaenoic acid (DHA), and n-3/n-6 ratio as well as the estimation of SCD16, SCD18 and ELOVL6 activities in RBC as independent predictors and C20:4n-6 (AA), C22:5n-3 (DPA), C22:6n-3 (DHA); or Omega-3 Index (O3I) (EPA + DHA, % total fatty acids) in RBC as dependent variables. Durbin–Watson (2.0 ± 0.4), collinearity (VIF < 5) and Shapiro–Wilk and Kolmogorov–Smirnov tests for normality were performed to test the validity of the models. The results are presented as mean ± SD values unless specified. The significance was set at *p* < 0.05.

## 3. Results

The characteristics of the study participants are summarized in Table 1. The sample population comprised subjects from both genders (males and females) with a mean age of 48.5 years and BMI of 27.8 kg/m^2^. A mean of 2483 ± 834 kcal/day intake was reported by the subjects, with proteins, carbohydrates and fat comprising 18.3 ± 2.6, 47.5 ± 8.4 and 34.6 ± 6.4, as energy percentage of dietary intake, respectively. The mean intake of SFAs and MUFAs was 10.1 ± 2.2 and 16.4 ± 4.3, respectively. The total PUFAs intake accounted for 5.2 ± 0.7% of energy. Those from n-6 PUFA and n-3 PUFA were 3.9 ± 0.9% and 0.6 ± 0.1%, respectively. The mean value (as percentage of fat) of OA, LA, AA, EPA and DHA in diet were 40.9; 11.1; 0.17; 0.16 and 0.37, respectively, with an n-3/n-6 ratio of 0.16 ± 0.05 (Table 1). Interestingly, in this sample population, DPA was the major n-3 HUFA found in the diet (Table 1). Indeed, Howe et al. [22] indicated that the presence of DPA in non-marine foods was mainly in meat products. This can help to explain the high level of DPA found in the dietary fat intake. In the present study, eggs, chicken, red meat and fatty fish were common constituents in consumed diets. The intake of these foodstuffs, considering the 3th quartile, was 22.2, 51.4, 51.4 and 42.7 g/day, respectively. The mean intake of milk, yogurt and cheese was 298, 135 and 29.4 g/day, respectively. The consumption of red meat accounted for 50 g/day approximately with a minimum of 8 g/day and a maximum of 120 g/day. Lean fish had a higher mean intake of 27 g/day compared to fatty fish (21.4 g/day) and salted cod (11.8 g/day). In the group of dietary fats, olive oil had the highest intake, reaching a mean value of 21 g/day.

**RBC membranes fatty acid profile.** The fatty acids profile (relative percentage) in red blood cell (RBC) membranes is listed in Table 1. SFAs were the most representative, and in this group, fatty acid C16:0 (PA) was 25.6 ± 1.8% of the total fatty acids. Isomers of C18:1, predominantly C18:1n-9 (OA), accounted for more than 13%. In the n-6 family, AA and LA were the most abundant, with 12.4% and 8.7%, respectively. Among n-3 HUFA, DHA was the most representative, accounting for 4.6 ± 1.0% (range 3.2–7.5), which was followed by DPA (1.3 ± 0.4%) and EPA (0.6 ± 0.3%). The mean value of the Omega-3 Index (O3I) obtained in RBC membranes was 5.3 ± 1.2%, ranging between 3.5 and 8.2 percent, which was in line with a study carried out in a Mediterranean population (mean age of 66 years and BMI 29.2), which found O3I values of 7.1% (6.08 to 8.05%) with a dietary EPA + DHA intake of 0.9 g/day [23].

**Correlations between dietary FA and RBC FA profile.** Both C16:0 (PA) and C18:0 (SA) in diet correlated negatively with n-3 HUFA and O3I in RBC. However, the effect seems to be more pronounced in the case of SA (Table 2). Oleic acid (OA) in diet had a significant and negative correlation with AA in RBC. Dietary EPA correlated significantly only with DPA in RBC membranes, and a positive association, that approaches statistical significance, was found with EPA (*p* = 0.056), DHA (*p* = 0.057) and O3I (*p* = 0.052). On the contrary, dietary DHA was correlated with all n-3 HUFA and O3I in RBC membranes (Table 2).

Using bivariate analysis, with the exception of the negative correlation between dietary SA and SCD18 (Table 2), no significant associations were found between FA intake and estimation of SDC16, SCD18 and ELOVL6 activities. Several nutritional and hormonal factors seem to be involved in SCD1 activity/expression. In general, dietary n-3 and n-6 FA decrease, while MUFA and mainly SFA increase *SCD1* expression by the direct and/or indirect regulation of SREBP-1c and/or liver X receptor (LXR) [24].

The positive correlation reported for SA in diet and PA in RBC remained significant after multi-adjustment and, although not significant (*p* = 0.057, *p* = 0.066 and *p* = 0.087), associated negatively with DHA, O3I and SCD18 in RBCs, respectively. Dietary DHA maintained the negative association with LA in RBC, and EPA and DHA in diet were positively associated with DPA in RBC membranes. Dietary oleic acid (OA) maintained the negative association with AA (*p* = 0.002) in RBC membranes, and the correlation was significantly stronger after multi-adjustment analysis. In addition, dietary OA also showed a negative and significant association with SA in RBCs (*p* < 0.05). Moreover, olive oil (comprising mainly by OA) negatively correlated with AA in RBCs (*p* = 0.039), which remained significant after multi-adjustment. Similar results were also observed by others using very-low density lipoproteins (VLDLs) as a biomarker. For instance, Perona et al. [25] in a subgroup of the PREDIMED study observed in the group supplemented with olive oil (Mediterranean Diet plus Virgin Olive Oil) a significant decrease in the level of AA in VLDL phospholipids.

**Associations between dietary fatty acids, RBC fatty acids and their ratios, and cardiometabolic biomarkers.** In the present work, by bivariate analysis, plasma hsCRP (median 0.94 mg/L) correlated positively with PA (*p* = 0.008) and negatively with estimation of ELOVL6 activity (*p* = 0.031) in RBC membranes. After multi-adjustment for BMI, age and total energy intake, only the association between hsCRP and PA in RBC remained significant, and both C18:1 and SCD18 (*p* < 0.05) showed significant and negative correlations with hsCRP.

As shown in Table 3, BMI positively correlated with estimation of SCD16 activity. SCD16 and SCD18 were positively associated with triacylglycerols (TAG) levels (Table 3), which is lost after multi-adjustment. In addition, SCD16 positively and significantly correlated with LDL-c and the LDL-c/HDL-c ratio and negatively with the ApoA1/ApoB ratio (Table 3), and it remained significant after multi-adjustment. By bivariate analysis, ELOVL6 was found to be negatively correlated with HDL-c (*p* = 0.056), although in the border value of significance, which became significant (*p* < 0.05) after multi-adjustment. In a subset of the population sample, SCD16 showed a negative and significant association with L-HDL subfraction (% and mg/dL) and positive with S-HDL (% and mg/dL) (Table 4). However, only the association with S-HDL subfraction (%) remained significant (*p* < 0.05) after multi-adjustment. Interestingly, ELOVL6 was found to be negatively and significantly associated with I-HDL (mg/dL) (Table 4), and it remained significant (*p* < 0.05) after multi-adjustment.

In the bivariate analysis, both EPA and DHA in diet negatively correlated with HDL-c levels, *p* = 0.026 and *p* = 0.017, respectively, which remained significant after multi-adjustment. PA in diet positively correlated with LDL-c, although not significantly (*p* = 0.071), and dietary LA exhibited a moderate-to-strong negative association with I-HDL (%) (rs = −0.726, *p* = 0.001) and remained significant (*p* = 0.003) after-multi-adjustment. Moreover, after multi-adjustment, a positive and significant correlation was found between OA in diet and I-HDL subfraction (mg/dL) (*p* < 0.05) and, although AA in diet showed a positive association with hsCRP plasma levels, this result lacks statistical significance (*p* = 0.097).

The positive correlations found between SCD16 and SCD18 with TAG plasma levels were in accordance with previously experimental data; for a review, see [24] and human studies [26]. The negative association between the estimation of ELOVL6 activity with HDL-c, that became statistically significant after multi-adjustment, is less obvious from a mechanistic point of view, but it may be related, at least partially, with an increase in the levels of unsaturated fatty acids (UFAs) that can lead to ATP-binding cassette transporter A1 (ABCA1) protein destabilization and, therefore, decreasing cholesterol efflux to HDL and/or changes in HDL particles biogenesis, as previously proposed for the relationships between SCD1 and HDL-c levels [24], or alterations in HDL particles remodeling. Furthermore, these data could explain the negative and significant association found between ELOVL6 and the I-HDL subfraction (Table 4) and in contrary to exogenously provided dietary oleic acid. In addition, the SCD16-associated high TAG/LDL-c levels phenotype may contribute to increased S-HDL and/or decreased L-HDL molecular subfractions that can be related to changes in HDL particles biogenesis/remodeling as well [27].

**Multivariate regression analysis.** With multivariate regression analysis, dietary OA was an independent predictor and negatively associated with AA levels in RBCs, and the estimation of ELOVL6 and SCD16 activities was positively associated with AA levels in RBCs (Table 5). Furthermore, OA in diet is positively associated with DPA in RBC (Table 6).

Interestingly, SA in diet was negatively correlated and ELOVL6 was positively associated with DHA (Table 7) and O3I (Table 8) in RBCs. Previously, researchers reported a positive association between higher proportions of SA and increased levels of n-6 FA (and/or higher n-6/n-3 ratio) in RBCs and risk of cognitive decline/loss in humans [28]. Moreover, in murine models, a high-fat diet rich in SA resulted in lower caloric energy expenditure, which was metabolically characterized by lower FA oxidation, increased adiposity, and hepatic insulin resistance [29].

The present data highlight the role of (patho)physiological mechanisms involved in the regulation of ELOVL6 activity/expression, which can also lead to changes in n-6/n-3 HUFA partition. In murine models and in vitro studies, ELOVL6 seems to be a key player in fatty acids metabolism in strict connection with SCD1/2, a major elongase for endogenously produced OA, and it was involved in the balance of C16:1/C18:1 positional isomers as well [30,31,32]. Interestingly, the hepatoprotective role of ursodeoxycholyl lysophosphatidylethanolamide (UDCA-LPE) observed in experimental models, such as anti-apoptotic, anti-inflammatory, or hypolipidemic effects, can be partially explained by an SCD1-mediated metabolic shift toward unsaturated fatty acids over cytotoxic SFA with an impact on TAG and PL levels and molecular composition [33]. These authors also indicated that UDCA-LPE, in murine hepatocytes, increased not only OA levels but also AA, DPA or DHA, which can be explained by the concomitant up-regulation of *FADS1 (D5D)*, *FAS*, *ELOVL6* and *PPARs* mRNAs [33].

## 4. Discussion

In the present study, by bivariate analysis, dietary oleic acid (OA) correlated negatively with C20:4n-6 (AA) in RBC, and with multivariate regression analysis, dietary OA was negatively associated with AA in RBCs, and the estimation of SCD16 and ELOVL6 activities was positively associated with AA in RBC.

Both dietary and endogenous synthesized oleoyl-CoA are preferential substrates for the production of new TAG, esterification of free cholesterol (CE), and the main MUFAs in phospholipids [10,11,34,35], such as phosphatidylcholine (PC). These lipid classes, mainly TAG and PC, can be assembled with ApoB100 to produce hepatic VLDL. SCD1 is the principal enzyme involved in the synthesis of endogenous MUFA (C16:1 and C18:1), and it is strongly related to de novo lipogenesis (DNL) [34]. Several transcription factors can regulate DNL, and lipogenic enzymes are involved in those pathways, such as fatty acid synthase (FAS) or acetyl-CoA carboxylase (ACC). SREBP1c and ChREBP are particularly important and are under regulation by dietary and hormonal factors. A carbohydrate-rich diet greatly stimulates DNL and SCD1 expression via ChREBP, and several dietary lipid components can also stimulate DNL and SCD1 expression via SREBP1c as well [10,11,34,35,36]. However, PUFAs, from both n-3 and n-6 families, are inhibitors of DNL and SCD1 directly and/or indirectly by regulating SREBP1c and ChREBP. It is currently accepted that the interplay between DNL and several elongases and desaturases with transcription factors is not only downstream but also upstream regulated as well [11,37,38]. These cross-regulation and feedback mechanisms can accurately sense the metabolic milieu and adjust several pathways, as it happens in changing the dietary pattern. In fact, SCD1 seems to be a central metabolic sensor involved in the correction of SFA/MUFA ratio [10,39,40]. When this regulatory process fails due to either acute or chronic events, the SFA/MUFA ratio is changed, and a cascade of events takes place [11].

Current Western diet (WD) patterns around the world are characterized by excessive energy-dense foodstuffs consumption, and they are rich in carbohydrates, fats and calories. This pattern can change endogenously the SFA/MUFA ratio by enhancing DNL and increasing the levels of SFA. It was demonstrated in Wistar rats that WD not only increased the SFA/MUFA ratio but also differentially changed the encoded genes for, e.g., *ELOVL6* and *SCD1* in a time-dependent manner [41]. The increase in SFA levels, mainly free palmitate, can lead to cell stress, e.g., endoplasmic reticulum (ER) stress, which in turn triggers unfolding protein response (UPR) [40]. The UPR, on one hand, increases the new biosynthesis of PL (Kennedy pathway) by favoring the translocation of mitochondrial cytidylyltransferase (CCT), which is a key enzyme in PC synthesis [39,40]. On the other hand, UPC favors the expression of LPCAT3, which is an important enzyme in the Lands pathway, with higher specificity for PUFAs, mostly n-6 PUFA, mainly AAs. Thus, cells avoid increasing free palmitate by esterification in lipid species and by increasing the SFA/MUFA ratio, maintaining the right balance in PL unsaturation by the incorporation of PUFAs, mainly AA. In *SCD1* knockdown cells, only LPCAT3 was significantly up-regulated [40], and PPARα seems to regulate the expression of this enzyme [42].

Therefore, free palmitate is well characterized by its citotoxic effect. The newly produced C16:0 (PA), from DNL, can undergo desaturation and was converted in 16:1, catalyzed by SCD16, and/or elongated, by ELOVL6 up to C18:0 (SA). SCD1 and ELOVL6 can also be regulated by each other [37]. On the other hand, SCD1 is also regulated post-translationally by microsomal degradation, which leads to a quick decay of the enzyme levels [10,11,34]. The hepatic expression of *ELOVL6* is activated by SREBP1 and suppressed by dietary PUFAs [37].

It is possible to accept that in case of increased availability of free palmitate, AA and eventually SA are good candidates as substrates for Kennedy and Lands pathways that incorporate both fatty acids in PL fractions. In the liver, LPCAT3 also shows preference for SA (re)acylation [43]. The geometric optimization B3LYP/6-31G(d,p) data for several *sn_1_*-*sn_2_*-PC molecular species with OA and AA in the *sn*_2_ position indicated that the more stable conformation, among all studied models, is observed in the case of 18:0-20:4n6-PC (Bispo et al., unpublished results). Pietiläinen et al. [44], suggested that the increase in AA in adipose tissue membranes of obese individuals compared to lean twins, although maintaining the biophysical properties of lipid membranes, can increase the vulnerability to inflammatory process as well. These authors also reported that ELOVL6 appears to have a pivotal role in this process, including a stronger association with SCD1, and that the knock-down of ELOVL6 leads to a reduction in PUFA-containing phospholipids [44]. Furthermore, Eto et al. [45], using C3H10T1/2 cells, reported an increase in, e.g., 16:0-20:4n6-PC and 18:0-20:4n6-PC molecular species, that accompany adipocytes differentiation as a result of increasing activity of LPLATs, mainly LPCAT3. Researchers observed a significant increase in FAS and ELOVL6 mRNAs (less obvious in the case of SCD mRNA) in the liver of obese subjects compared with visceral abdominal tissue [46].

Both PLA2 and LPCATs have been shown to strongly regulate the level of cellular content in AA [47]. In resting cells (unstimulated), the reacylation reactions dominate, maintaining free AA at low levels. However, after stimulation, the level of free AA is found in a higher amount, and it can undergo COX and LOX mediated-eicosanoid production [48,49]. It was proposed that in response to ER stress, cluster of differentiation 36 (CD36) can mediate the release of AA from cellular membranes generating pro-inflammatory eicosanoids [50]. Interestingly, higher levels of stearoyl-lysophosphatidylcoline and lower levels of oleoyl-lysophosphatidylcoline were found in patients with cancer, and in an animal model of ulcerative colitis, a severe inflammatory bowel disease [38,51].

Dietary OA can increase the availability of oleoyl-CoA, leading to a decrease in the SFA/MUFA ratio that is sensed by cells. On the other side, OA from diet can compete with AA for LPCAT3 (re)acylation of PC and also for several other important proteins involved in FA metabolism, like long-chain acyl-coenzyme A synthetase (e.g., ACSL4) or fatty acid-binding proteins (FABP) [43,47]. Moreover, dietary OA can increase the substrate available for the neosynthesis of TAG and CE, leading to the incorporation of, e.g., free palmitate in these lipid species that reduces UPR and ER stress, and it can normalize new PL biosynthesis, mainly PC [37]. Although with conflicting results, OA, on contrary to PUFAs, can induce ApoB100 secretion [52] that is associated with the decrease in several ER stress markers. In addition, Green et al. [32] also suggest that MUFAs (mainly endogenously produced) can provide negative-feedback control on *ELOVL6* expression. In the present work, despite no significant correlation being observed between dietary OA and the estimation of SCD1 or ELOVL6 activities (*p* > 0.05), dietary OA negatively correlated with SA (C18:0) in RBCs (*p*< 0.05), after multi-adjustment analysis. Higher levels of SA were previously linked to increased membrane destabilization and loss of fluidity [53]. Deguil et al. [54] reported that in *hem1* cells, exogenously UFA are incorporated into PLs and directly regulate the properties of membranes creating a more disordered bilayer, which in turn can also modulate SFA-induced UPR “in vivo”. Furthermore, such an effect depends on several factors, such as acyl chain length or degree of unsaturation [54]. These authors also demonstrated that among several UFAs, OA (C18:1n-9) seems to create a perfect width in PL biomembranes that can avoid the hydrophobic mismatch, andthat UFA increases the cross-sectional area of the acyl chain region in PC molecular species [54]. In addition, Pietiläinen et al. [44], using molecular modeling simulations, reported that enhancing PUFA-rich PC molecular species (and also the PC/PE ratio) increased the area “per” lipid and consequently the fluidity of biomembranes. These findings can underline and explain the aforementioned increased levels of AA in adipose membranes of obese subjects despite their lower dietary PUFAs intake [44].

Several lipidic ligands have been described to react with PPARα. The first endogenous ligand for PPARα has been identified and the most effective seems to be the 1-palmitoyl-2-oleoyl-glycerol-3PC [55]. Interestingly, it was found that the activation/expression of lipogenic enzymes, mainly FAS, is determinant for endogenous ligand formation. Fibrates, such as clofibric acid (and/or derivatives), a PPARα agonist, that increase SCD1 transcription and protein half life [24], by delaying the degradation rate, can also regulate LPCAT(3), AAC, or ELOVL6 in experimental models, that are supplemented by the increase in liver C18:1 mass production and incorporation in the sn2 position of PC, which is concomitant with the reduction in PUFA levels, mainly LA (C18:2n-6), observed in both animal models and humans [56,57,58]. These data support the fact that a highly metabolic regulation is necessary to maintain the proper endogenous balance of SFA/MUFA ratio to sustain cell integrity and to protect from PA (C16:0) lipotoxicity, and that PPARα is an important player in sensing cells’ molecular milieu. The dimerization PPARα-LXR can modulate several other pathways involved in lipid and energy homeostasis, such as SCD1, and that PPARα increases several enzymes involved in lipid oxidation [10,37]. Furthermore, several fatty acids, particularly PUFAs, are important ligands for PPARs [59].

Based on the correlations found in the present study, the intake of dietary OA (mainly from olive oil) can have a synergic effect with the simultaneous intake of n-3 HUFAs, which is a characteristic feature of the Mediterranean diet profile [60]. These fatty acids can compete with n-6 PUFAs, such as AA, for (re)acylation in membranes and function as a substrate for COX and LOX enzymes. Previous data, using isocaloric diets with graded amounts of n-3 HUFA, in the Wistar rat model, pointed out the importance of a diet with canned sardines in several tissues’ biomembranes, including RBCs, which are responsible for protective lipid mediators and anti-inflammatory effect [61]. For instance, DHA can reverse and/or attenuate the pro-inflammatory effect of free palmitate mediated by toll-like receptors (TLR)/inflammasome, and it reduced lipid accumulation and inflammation by suppressing NOD-like receptors and caspase-1 activation in HepG2 cells [62], which is a mechanism involved in the cardiometabolic effect associated with the metabolic syndrome. Additionally, n-3 HUFA, mainly EPA [63], and OA by regulating several signaling/transcription pathways [64] can increase fatty acid oxidation. Interestingly, recent findings in individuals at risk of metabolic syndrome demonstrate that the MUFA-rich diet (from olive oil) is responsible for an antiatherogenic lipid profile [65]. In the present work, a positive and significant correlation was found between OA in diet and only I-HDL subfraction. SCD16 positively and significantly correlated with LDL-c and the LDL-c/HDL-c ratio and negatively with the ApoA1/ApoB ratio. ELOVL6 was found to be negatively correlated with HDL-c, although in the border value of significance, which became significant (*p* < 0.05) after multi-adjustment. In a subset of the population sample, SCD16 showed a negative and significant association with the L-HDL subfraction and a positive association with S-HDL, and the latter seems to be less cardioprotective. Both SCD16 and SCD18 positively correlated with TAG blood levels, which is a metabolic feature of metabolic syndrome. A recent study in individuals with metabolically healthy (MH) and unhealthy (MU) phenotypes reported that individuals with MH had lower estimated SCD16 and SCD18 activities [66].

In addition, a very recent study, in model cells, concluded that OA exerted anti-inflammatory effects by acting to inhibit the NFκB pathway [67]. OA can also exert a protective effect against the very-long-chain fatty acids-induced apoptosis in peroxisome-deficient CHO cells, which may be related with ameliorating the ER and membrane stress [68].

The present work has some limitations. Firstly, there was a small number of individuals. The participants also had a high consumption of OA, mainly from olive oil, which does not allow generalization. Thus, there is a need for future work with a larger sample population.

## 5. Conclusions

The present study showed that OA, SA, in diet, and estimated activities of SCD16 and ELOVL6 are independent predictors of n-6 and n-3 HUFA in RBC membranes. From the above-mentioned results and mechanistic considerations, dietary OA can directly and/or indirectly influence the levels of AA in RBC membranes, which, in turn, can be used as a proxy model for several other cells and tissue “in vivo”. By modifying the levels of n-6 and n-3 HUFA in biomembranes’ PL, dietary OA can favor a beneficial biochemical environment that prudently induce a protective production of anti-inflammatory mediators and signaling molecules. Taking this into account, these results reinforce the importance of the Mediterranean diet, particularly the intake of dietary oleic acid, which is the main fatty acid of olive oil.

## Figures and Tables

**Table 1:** Summary of participants’ features, RBC fatty acids and dietary components.

	Mean ± SD
**Clinical characteristics**	
Age (y)	48.5 ± 18.5
BMI (Kg/m^2^)	27.8 ± 4.3
**Biochemical characteristics**	
Glucose (mg/dL)	104.5 ± 39.6
Total cholesterol (mg/dL)	187.9 ± 43.5
LDL-c (mg/dL)	115.8 ± 40.0
Triacylglycerols (mg/dL)	105.4 ± 58.9
HDL cholesterol (mg/dL)	51.0 ± 9.5
LDL-c/HDL-c	2.3 ± 0.9
ApoAI/ApoB	1.8 ± 0.5
**RBC fatty acids (% total fatty acids) and ratios**	
C16:0	25.6 ± 1.8
C16:1	0.37 ± 0.17
C18:0	15.7 ± 0.5
C18:1	13.1 ± 0.9
C18:2 (n-6)	8.7 ± 1.6
C20:4 (n-6)	12.4 ± 0.9
C20:5 (n-3)	0.6 ± 0.3
C22:5 (n-3)	1.3 ± 0.4
C22:6 (n-3)	4.6 ± 1.0
EPA + DHA (O3I) *	5.3 ± 1.2
SCD16	0.014 ± 0.006
SCD18	0.84 ± 0.06
ELOVL6	0.62 ± 0.05
**Daily nutrient intake**	
Energy (Kcal)	2483 ± 834
Proteins (%E)	18.3 ± 2.6
Carbohydrates (%E)	47.5 ± 8.4
Fat (%E)	34.6 ± 6.4
Cholesterol (mg)	334.3 ± 123.6
n-3/n-6	0.16 ± 0.05
Palmitic acid (% Fat)	15.6 ± 1.5
Stearic acid (% Fat)	6.1 ± 0.96
Oleic acid (% Fat)	40.9 ± 5.4
Linoleic acid (% Fat)	11.1 ± 2.1
Arachidonic acid (% Fat)	0.17 ± 0.06
Eicosapentaenoic acid (% Fat)	0.16 ± 0.11
Docosapentaenoic acid (% Fat)	0.46 ± 0.42
Docosahexaenoic acid (% Fat)	0.37 ± 0.25

* O3I: Omega-3 Index.

**Table 2:** Bivariate correlations between dietary fatty acids and RBC membranes fatty acids, and estimation of SCD16, SCD18 and ELOVL6 activities.

RBC Fatty Acids (%) and Their Ratios													
	C16:0	C18:0	C16:1	C18:1	C18:2n-6	C20:4n-6	C20:5n-3	C22:5n-3	C22:6n-3	O3I	SCD16	SCD18	ELOVL6
*Diet (% Fat)*													
C16:0	0.558 ^b^	*ns*	*ns*	*ns*	*ns*	*ns*	*ns*	−0.570 ^b^	−0.467 ^a^	−0.449 ^a^	*ns*	*ns*	*ns*
C18:0	0.594 ^b^	*ns*	*ns*	*ns*	0.445 ^a^	*ns*	−0.470 ^a^	−0.613 ^b^	−0.534 ^a^	−0.541 ^a^	*ns*	−0.442 ^a^	*ns*
C18:1n-9	*ns*	*ns*	*ns*	*ns*	*ns*	−0.470^a^	*ns*	*ns*	*ns*	*ns*	*ns*	*ns*	*ns*
C18:2n-6	*ns*	*ns*	*ns*	*ns*	*ns*	*ns*	*ns*	*ns*	*ns*	*ns*	*ns*	*ns*	*ns*
C20:4n-6	*ns*	*ns*	*ns*	*ns*	*ns*	*ns*	*ns*	*ns*	*ns*	*ns*	*ns*	*ns*	*ns*
C20:5n-3	*ns*	*ns*	*ns*	*ns*	−0.503 ^a^	*ns*	*ns*	0.581 ^b^	*ns*	*ns*	*ns*	*ns*	*ns*
C22:6n-3	*ns*	*ns*	*ns*	*ns*	−0.545 ^a^	*ns*	0.466 ^a^	0.613 ^b^	0.477 ^a^	0.484 ^a^	*ns*	*ns*	*ns*

^a^ *p* < 0.05; ^b^ *p* ≤ 0.01; *ns*: not significant.

**Table 3:** Bivariate correlations between estimation of SCD16, SCD18 and ELOVL6 activities and age, BMI and cardiometabolic biomarkers.

	AGE	BMI	Glucose	HDL-c	Total-Chol	TAG	ApoA1/ApoB	LDL-c	LDL-c/HDL-c
SCD18	*ns*	*ns*	0.601 ^b^	*ns*	*ns*	0.446 ^a^	*ns*	*ns*	*ns*
SCD16	*ns*	0.446 ^a^	*ns*	*ns*	0.575 ^b^	0.483 ^a^	−0.566 ^b^	0.605 ^b^	0.616 ^b^
ELOVL6	*ns*	*ns*	*ns*	*ns*	*ns*	*ns*	*ns*	*ns*	*ns*

^a^ *p* < 0.05; ^b^ *p* ≤ 0.01; *ns*: not significant.

**Table 4:** Bivariate correlations between estimation of SCD16, SCD18 and ELOVL6 activities and HDL molecular subfractions (*n* = 16).

	L-HDL (%)	I-HDL (%)	S-HDL (%)	L-HDL (mg/dL)	I-HDL (mg/dL)	S-HDL (mg/dL)
SCD18	*ns*	*ns*	*ns*	*ns*	*ns*	*ns*
SCD16	−0.562 ^a^	*ns*	0.731 ^c^	−0.512 ^a^	*ns*	0.594 ^a^
ELOVL6	*ns*	*ns*	*ns*	*ns*	−0.606 ^a^	*ns*

^a^ *p* < 0.05; ^c^ *p* ≤ 0.001; *ns*: not significant.

**Table 5:** Multivariate regression analysis (stepwise method) for C20:4n-6 (AA) in RBC as dependent variable.

		AA-RBC (%)		
		*Model*	R^2^ adjust	*p* value
			0.606	<0.001
	Beta adjusted			
C18:1n-9 diet	−0.623			<0.001
ELOVL6	0.458			<0.01
SCD16	0.369			<0.05

**Table 6:** Multivariate regression analysis (stepwise method) for C22:5n-3 (DPA) in RBC as dependent variable.

		DPA-RBC (%)		
		*Model*	R^2^ adjust	*p* value
			0.478	0.001
	Beta adjusted			
C18:1n-9 diet	0.400			<0.05
C20:5n-3 diet	0.664			0.001

**Table 7:** Multivariate regression analysis (stepwise method) for C22:6n-3 (DHA) in RBC as a dependent variable.

		DHA-RBC (%)		
		*Model*	R^2^ adjust	*p* value
			0.469	0.001
	Beta adjusted			
C18:0 diet	−0.478			<0.05
ELOVL6	0.405			<0.05

**Table 8:** Multivariate regression analysis (stepwise method) for omega-3 index (O3I) in RBC as a dependent variable.

		O3I-RBC (%)		
		*Model*	R^2^ adjust	*p* value
			0.429	<0.01
	Beta adjusted			
C18:0 diet	−0.462			<0.05
ELOVL6	0.390			<0.05

## Data Availability

Data are contained within the article.
